# The Respiratory Syncytial Virus G Protein Conserved Domain Induces a Persistent and Protective Antibody Response in Rodents

**DOI:** 10.1371/journal.pone.0034331

**Published:** 2012-03-29

**Authors:** Thien N. Nguyen, Ultan F. Power, Alain Robert, Jean-François Haeuw, Katia Helffer, Amadeo Perez, Miguel-Angel Asin, Nathalie Corvaia, Christine Libon

**Affiliations:** 1 Microbiotechnologie, Centre de Recherche and Développement Pierre Fabre, Toulouse, France; 2 Medical Biology Centre, Centre for Infection and Immunity, School of Medicine, Dentistry and Biomedical Sciences, Queen's University Belfast, Belfast, Northern Ireland; 3 Centre d'Immunologie Pierre Fabre, St-Julien-en-Genevois, France; 4 Centre de Recherche Galénique Pierre Fabre, Barcelona, Spain; University of Iowa, United States of America

## Abstract

Respiratory syncytial virus (RSV) is an important cause of severe upper and lower respiratory disease in infants and in the elderly. There are 2 main RSV subtypes A and B. A recombinant vaccine was designed based on the central domain of the RSV-A attachment G protein which we had previously named G2Na (aa130–230). Here we evaluated immunogenicity, persistence of antibody (Ab) response and protective efficacy induced in rodents by: (i) G2Na fused to DT (Diphtheria toxin) fragments in cotton rats. DT fusion did not potentiate neutralizing Ab responses against RSV-A or cross-reactivity to RSV-B. (ii) G2Nb (aa130–230 of the RSV-B G protein) either fused to, or admixed with G2Na. G2Nb did not induce RSV-B-reactive Ab responses. (iii) G2Na at low doses. Two injections of 3 µg G2Na in Alum were sufficient to induce protective immune responses in mouse lungs, preventing RSV-A and greatly reducing RSV-B infections. In cotton rats, G2Na-induced RSV-reactive Ab and protective immunity against RSV-A challenge that persisted for at least 24 weeks. (iv) injecting RSV primed mice with a single dose of G2Na/Alum or G2Na/PLGA [poly(D,L-lactide-co-glycolide]. Despite the presence of pre-existing RSV-specific Abs, these formulations effectively boosted anti-RSV Ab titres and increased Ab titres persisted for at least 21 weeks. Affinity maturation of these Abs increased from day 28 to day 148. These data indicate that G2Na has potential as a component of an RSV vaccine formulation.

## Introduction

RSV is a viral pathogen causing a range of symptoms from mild upper to severe lower respiratory tract infection in infants, in immunocompromised individuals and in the elderly [1], [2]. Every year in the US, 75,000–125,000 children <1 year old are hospitalised and an estimated 250 deaths occur, due to RSV infection. It is responsible for 1.5 million outpatient visits among children aged <5 years annually in the US [3]. Natural RSV primary infections do not confer complete protection. Despite many decades of research and development, an RSV vaccine is still not available.

**Figure 1:**
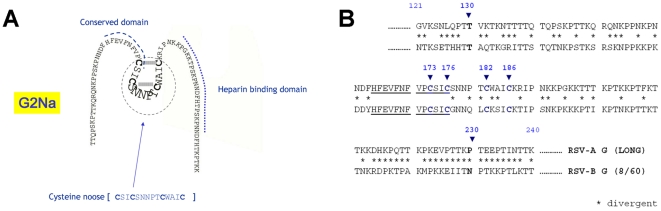
Central conserved domain of RSV G attachment protein (aa130–230). We present here the aa sequence of the conserved domain of (A) G2Na in a hypothetical schematic 2D structure with the highly conserved 13 aa domain followed by the Cysteine noose (dotted circle) [sequence in brackets with Cys res. in bold]. Note the disulphide bridge between the Cys res. and the Lys rich heparin binding domain. (B) G2Na derived from RSV-A (Long) G protein compared to G2Nb derived from RSV-B (8/60) G protein from aa121–240. Conserved domains are underlined. Cys res. positions in bold letters are respectively 173, 176, 182 and 186. (* divergent).

The formalin-inactivated RSV vaccine tested in field trials in the 1960s caused enhanced disease in vaccinated infants. These factors have greatly complicated the development of a safe and efficacious RSV vaccine [4]. Research and development of immunoprophylaxis (vaccines and humanised monoclonal Ab) against RSV were recently reviewed by Power [5], Graham [6], van Bleek [7] Chang [8] and Collins [9]. RSV F and G glycoproteins are the protective antigens inducing neutralizing Ab against RSV infection. Both F and G proteins and peptides have been subjected to numerous clinical trials. Among them, G2Na, the central conserved region of RSV-A attachment glycoprotein G (aa130–230) (Fig. 1A & 1B) has been tested in the subunit vaccine candidate BBG2Na [10], [11], [12]. G2Na was C-terminally fused to BB, the albumin binding domain of Streptococcal protein G. BBG2Na reached clinical phase III but was halted due to rare adverse events in a very small number of vaccinees. The chronology of the events, the delay to onset and the symptoms were suggestive of a vaccine-related type III hypersensitivity-like reaction. When tested in a rabbit model of type III hypersensitivity, we found that BBG2Na induced an Arthus reaction and that the BB component, rather than G2Na, was responsible [13]. This provided the impetus for further studies on the immunogenicity and protective efficacy of G2Na as an RSV vaccine component. This non-glycosylated fragment includes a 13 aa peptide that is completely conserved between all known A and B strains, a correctly linked cysteine noose formed by 4 conserved Cys residue (res.) [14], a heparin binding domain [15] and a CX3C chemokine motif [16]. G2Na contains 5 human B cell epitopes (2 of which overlap) called protectopes [17], [18], including a highly immunogenic peptide (G174–187) [19], and a Th epitope (G184–198) [20]. The conserved Cys res. play an important role in the enhancement of CTL responses towards other RSV antigens (Ag) [21]. Peptides incorporating the 5 protectopes (coupled to carrier protein P40, the outer membrane protein A from *Klebsiella pneumoniae*) induced protective immune responses against RSV-A challenge [17]. Passive-transfer of Mabs (generated against the central domain G peptides) protect animal lungs from RSV challenge without enhanced immunopathology [22], [23], [24]. Furthermore, immunization with G peptides or protein spanning the central conserved region induced Abs that block the CX3C-CX3CR1 interaction of RSV G protein and reduced pulmonary inflammation and virus replication in mice [25].

In this study, we documented in rodents the immunogenicity and protective efficacy of the 11.8 KDa G2Na or G2Nb (the RSV B-derived equivalent of G2Na), genetically fused or not with carrier proteins. The vaccine candidates were variously formulated in Alum or PLGA and compared. In particular, we report the induction and duration of protective immunity following vaccination and heterologous protective immunity against RSV B. Furthermore, in a mouse model mimicking pre-existing RSV immunity, we determined the capacity of G2Na vaccine formulations to boost and cause affinity maturation of the Ab response following live RSV priming. Our data provide the rationale for further studies of G2Na as an RSV vaccine component.

## Materials and Methods

### Viruses, cells, viral ELISA antigens

RSV-A Long strain (ATCC VR-26, American Type Culture Collection, Rockville, MD), and strain BT2a, (derived from an infant hospitalised with bronchiolitis and isolated in Dr. Power's laboratory) as well as RSV-B strain 8/60 (kindly provided by Dr. G. Taylor, Institute of Animal Health, Compton, UK) and strain CH18537 (kindly provided by Prof. Geoff Toms, University of Newcastle-upon-Tyne, UK) were propagated in HEp-2 cells (ECACC 86030501, European Collection of Animal Cell Cultures, Porton Down, Salisbury, UK) as previously described [19], except that DMEM was supplemented with 1% fetal calf serum. Viruses were harvested when extensive cytopathic effect was evident, by scraping attached cells into the medium. Purified viral antigens were generated by ultra-centrifugation of stocks of each virus on a discontinuous sucrose gradient, pelleting purified virions and resuspending the pellets in 0.5% (v/v) IGPAL (Sigma Aldrich, UK) in H_2_O. The protein content of the virus ELISA antigen stock was measured using the NanoOrange Protein Quantification kit (Molecular Probes, Invitrogen) and an appropriate protein dilution to be used in the ELISA assays were determined in a checker board assay.

### Gene constructs, production and purification of recombinant proteins

**Figure 2:**
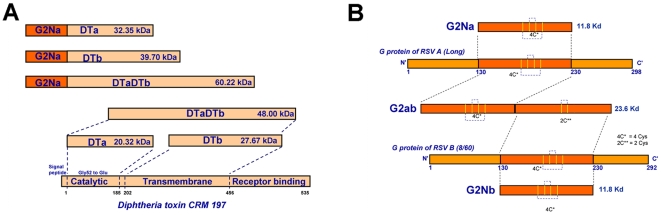
Schematic presentation of recombinant G protein derivatives. The gene encoding G2Na was fused to (A) DT derivatives resulting in G2NaDTa, G2NaDTb and G2NaDTaDTb. DTa was subcloned the nucleotide sequence encoding (aa1–185) of the catalytic domain of DT as described in Materials and Methods. Note that Gly res. at aa52 was substituted by Glu in order to inactivate DT toxicity. DTb was subcloned from the nucleotide sequence encoding (aa202–456) of the transmembrane domain of DT. DTaDTb is a fusion product of DTa to DTb. (B) G2b (aa130–230) of RSV-B G protein (8/60 strain) resulting in G2ab: in this fusion form, G2Na has 4 conserved Cys but G2b has only 2 conserved Cys176 and 182, the 2 outer Cys173 and Cys186 were substituted by Ser res. in order to avoid spurious disulphide bridges. G2Nb used for admixing with G2Na has all four Cys res.

For the first experiment, we designed 3 fusion molecules G2NaDTa, G2NaDTb, G2NaDTaDTb where: DTa (aa1–185) the A subunit of DT where res. Gly 52 was substituted by Glu, this single change was shown to deactivate the toxicity of DT namely CRM 197 [26]; DTb (aa 202–456) is the transmembrane domain B subunit of DT and DTaDTb is the fusion molecule of both (Fig. 2A). DT, in particular CRM 197, is commonly used as carrier protein for non immunogenic Ag like polysaccharides (conjugate vaccine) in licensed human vaccines. For comparison, we also included in this experiment BBG2Na, where BB (albumin binding domain of Streptococcal protein G) was characterised as a carrier protein [27]. For the 2^nd^ experiment, G2Nb was designed similarly to G2Na (Fig. 2B) from aa 130 to 230 of RSV-B (8/60 strain) G protein comprising the 13 aa conserved peptides and the 4 Cys conserved res.. In the fusion protein G2ab, G2Na cysteine noose was kept intact (Fig. 1A) but in the G2b cysteine loop region, the 2 outer Cys (C173 and C186) were substituted by Ser res. (Fig. 2B). This was designed to minimise non-desired inter or intra disulfide bridges which could alter the conformational structure of the cysteine noose. Recombinant Ags were expressed in *E. coli*: G2Na, G2NaDTa, G2NaDTb, G2NaDTaDTb, G2Nb, G2ab and BBG2Na. Genes encoding G2Na and G2Nb were assembled using synthetic oligonucleotides designed with optimised usage codon for *E. coli*, according to Nguyen [28]. In G2ab where the 2 Cys (C173 and C186) res. of the G2Nb were substituted by Ser res., we used PCR-mutagenesis to change Cys res. into Ser res. The 2^nd^ step was gene fusion to G2Na to generate a gene encoding G2ab. Genes encoding DTa and DTb were obtained by PCR directly on *Corynebacterium diphtheriae* genomic DNA (ATCC, Rockville, MD). Site directed PCR-mutagenesis was performed to change Gly (aa52) into Glu in the DTa. Gene constructs encoding G2NaDTa, G2NaDTb, G2NaDTaDTb were subcloned in a *Trp* based expression vector. The recombinant protein antigens were produced in *E.coli* ICONE cells and recovered as inclusion bodies, before extraction, refolding and purification [29]. These proteins were purified via the His Tag on an IMAC column with high yield and high purity. Clinical grade G2Na and BBG2Na were obtained after purification to homogeneity by a five chromatography step procedure including anion exchange, size exclusion chromatography and hydrophobic interaction chromatography. BBG2Na and G2Na primary structures were confirmed by ES-MS (Electrospray Mass spectrometry) [14].

### Antigens formulations in Alum or in PLGA

Antigens were adsorbed to Alum (Alhydrogel or Adju-Phos, Superfos BioSector, Danmark) in PBS before injections. In each experiment the quantity of each fusion protein was calculated as equivalents in molarity amounts to G2Na.

For the last experiment, biodegradable polymeric particles were used to encapsulate G2Na antigen. They represent an exciting approach to control the release of vaccine antigens to reduce the number of injections in the immunization schedule and optimise the desired immune response via selective targeting of antigen to antigen presenting cells [30], [31]. The hypothesis was based on the idea that intermittent intra-corporal release of antigen over a period of months might periodically boost the RSV-specific immune responses and thereby maintain elevated antibody titres over a longer period. G2Na-loaded PLGA microspheres were prepared by water/oil/water emulsion/solvent evaporation method using a 75∶25 PLGA copolymer (Boehringer Ingelheim), intrinsic viscosity of about 0.6 dL/g, which contains alkyl ester end groups. 173.3 ml of methylene chloride were added to 25.5 g of PLGA. 23 ml of a concentrated solution of G2Na (18 mg/ml) in a phosphate buffer solution (pH 7.5) were added to the PLGA organic solution and emulsified by sonication. This primary emulsion was added using a pump to an external phase consisting of an aqueous solution of 8% (w/v) polyvinyl pyrrolidone K90, 0.37 (v/v) polysorbate 80 and 1% (w/v) sodium chloride and mixed in order to emulsify. A volume of purified water was added and the methylene chloride was allowed to evaporate by bubbling. The resultant microspheres were sieved through a 90 µm mesh, collected by filtration, washed 3 times, and then freeze dried (Fig. S1). The average diameter of the microspheres was 14.8 µm, while microencapsulation efficiency was 68%. Blank microspheres were prepared by o/w emulsion/solvent evaporation method using a 75∶25 PLGA copolymer, intrinsic viscosity of about 0.6 dL/g, which contains alkyl ester end groups. Controls: after mild dissolution of G2Na-loaded PLGA microspheres with acetone followed by precipitation of the proteins, it was found that the majority of microencapsulated G2Na was recovered (>85%). By ELISA assays using the G2Na-specific Mab 18D1, which recognises the cysteine noose protectope [17], it was found that about 90% of the incorporated protein were structurally well-conserved.

### Animals and Immunisations

Female BALB/c mice, aged 6–9 weeks, were purchased from IFFA CREDO (L'Arbresle, France) and kept under specific pathogen-free conditions. They were fed with mouse maintenance diet A04 (UAR, Villemoissin-sur-Orge, France) and water *ad libitum*, and were housed and manipulated according to French and European guidelines. Five to eight week old male cotton rats (*Sigmodon hispidus*) were purchased from Virion Systems Inc. (Rockville, MD, USA), housed individually and kept under specific pathogen-free conditions. The cotton rats received sterile food and water *ad libitum* and were housed and manipulated according to French, UK and European guidelines. Mice and cotton rats were confirmed seronegative vis-à-vis RSV before inclusion in the studies.

Animals were immunised intraperitoneally (*ip*) with a 200 µl volume, or intramuscularly (*im*) with a 100 µl volume, in PBS containing 20% Alhydrogel or Adju-Phos. Palivizumab (Synagis®, Abbott Lab.), a humanised monoclonal antibody specific for the RSV F protein, is the only RSV-specific product on the market and is used prophylactically [32]. Palivizumab was used at 2 doses: 1.25 mg/Kg and 5 mg/Kg in PBS. Cotton rats were injected *im* with palivizumab one day before RSV challenge.

### ELISA assays and virus titration

RSV-A strains included Long (ATCC VR-26) and BT2a (clinical isolate). The RSV-B strains included CH18537 and 8/60. As indicated in each experiment, animals were bled according to the experimental schedule. G2Na-, G2Nb-, RSV-A- and RSV-B-specific IgG titres were determined by ELISA as previously described [10] using goat anti-mouse IgG-HRP conjugate (Jackson Immuno Research Laboratories, Baltimore, MD) or rabbit anti-cotton rat IgG (Virion Systems Inc., USA) followed by HRP-conjugated affinity purified goat anti-rabbit IgG (ICN). Mouse ELISA titres were expressed as the reciprocal of the last dilution with an OD (450 nm) at least twofold that of the negative control. A positive control serum (heat-inactivated at 56°C for 30 min) from cotton rats immunised with G2Na formulated in TiterMax Gold were included in all ELISA plates. Rat ELISA titres are referred to as arbitrary units and are calculated on the basis of regression analysis in relation to the positive control serum serial dilution curve, in which the first dilution is arbitrarily assigned a value of 10,000. Immunised animals were challenged with 10^5^ tissue culture infectious doses_50_ (TCID_50_) RSV-A or 10^6^ (TCID_50_) RSV-B by intranasal (*in*) installation and sacrificed 5 days post-challenge. To determine protective efficacy, animals were anesthetised and exsanguinated by cardiac puncture. Lung removal, lung homogenate preparation and virus titrations were undertaken as previously described [10]. The limit of detection for lung tissues was ≤1.45 log_10_ TCID_50_/g of lung, except when insufficient lung homogenate was available to inoculate 4 wells with undiluted homogenate, in which case the detection limit was determined as a function of the number of wells (1–3) inoculated with undiluted homogenates. Animal lungs were considered protected when mean virus titres were reduced by at least 2 log_10_ relative to PBS-immunised control animals.

### Avidity determination

Avidity of anti-RSV-A IgG Ab was determined by an ELISA elution assay using thiocyanate (NH_4_SCN) as a chaotropic agent (Sigma, St Louis, MO)[33]. Sera were pre-titrated and adjusted by dilution to a concentration giving ELISA results in the upper portion of the linear part of the standard curve (OD (450 nm) approx. 1.5). Following incubation of serum dilutions, NH_4_SCN was added at a final concentration ranging from 0 to 6 M (mole/L). Plates were then incubated for 15 min at room temperature, prior to the addition of the goat anti-mouse IgG-HRP conjugate and finally HRP substrate. The amount of Ab remaining bound to the plate, at each NH_4_SCN concentration, was calculated in units by reference to the ELISA standard curve. The avidity index (AI), corresponding to the concentration of thiocyanate required to elute 50% of the Ab units, was calculated. Low avidity Ab were defined by the fraction eluted at <3 M NH_4_SCN concentrations, whereas high-avidity IgG Ab were defined as those only eluted at ≥3 M NH_4_SCN concentrations according to [49].

### Statistical analyses

Antibody titres were analysed using SigmaStat® 3.5 statistical software using ANOVA followed by Holm-Sidak test on log10 values. Results were considered statistically significant if p<0.05.

### Ethics statement

Procedures and experiments involving mice and cotton rats were performed in accordance with French legislation under personal licence number 74-16 and this work was performed under the agreement C 74-243-8 obtained from the “Direction Départementale des services vétérinaires de Haute-Savoie”. The experiments on cotton rats (chapter duration of protective immunidy induced by G2Na in cotton rats) were performed under personal licence number PIL982b and project licence PPL2595b, both issued by the Northern Ireland Department of Health, Social Services and Public Safety, pursuant to the Animals (Scientific Procedures) Act 1986.

## Results

### Immunogenicity and protective efficacy conferred by G fragment constructs in cotton rats

**Figure 3:**
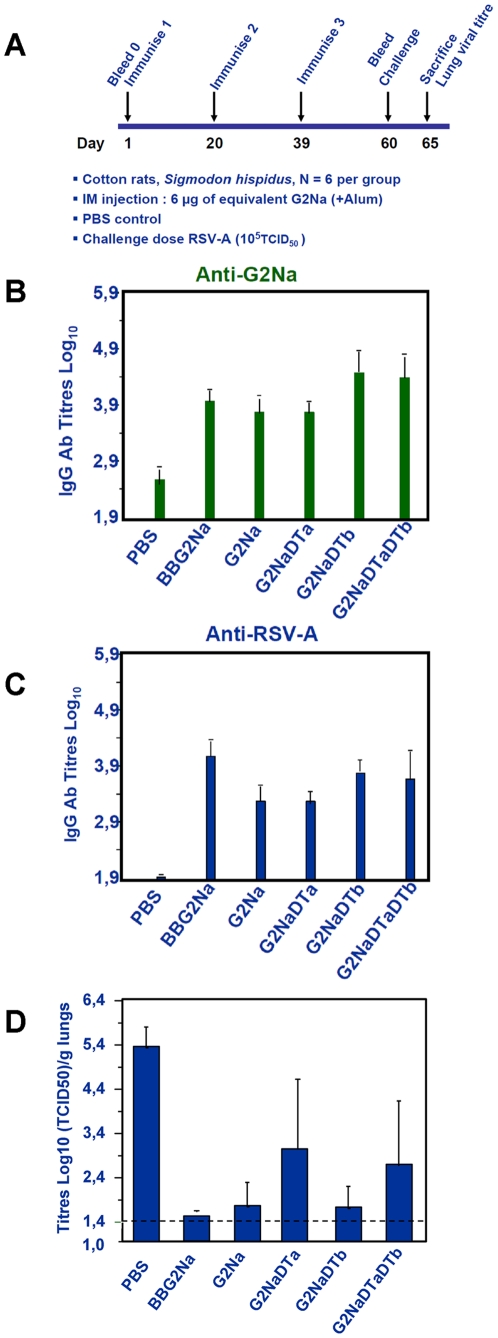
Immunogenicity and protective efficacy against RSV-A conferred by G2Na, BBG2Na, G2NaDTa, G2NaDTb and G2NaDTaDTb in cotton rats. Groups of animals were immunised thrice *im* with G2Na derivatives according to the outlined experimental protocol (A). After the 3^rd^ injection, on D60, we determined by ELISA the (B) anti-G2Na IgG Ab titre, (C) anti-RSV-A (Long) IgG Ab titre. Five days after challenge with RSV-A (10^5^ TCID_50_) we evaluated protective efficacy by determining the RSV-A titre in the lungs of immunised animals. Data were expressed as Log_10_ TCID_50_/g lung tissue. The dotted line represents the detection limit of RSV-A in the lungs.

G2Na is the RSV G protein component of the well characterised RSV vaccine candidate BBG2Na [10], [11]. However, there is limited information concerning the vaccine potential of G2Na without BB. In view of the adverse events encounter with BBG2Na in Phase III clinical trials, and the demonstration that the BB component was likely responsible for them, we generated a series of constructs incorporating G2Na and diphtheria toxin derivates as fusion carrier protein partners with a view to producing a novel RSV vaccine candidate. The constructs included G2NaDTa (G2Na N-terminal fused to Diphtheria subunit A, DTa), G2NaDTb (G2Na N-terminal fused to Diphtheria subunit B, DTb) and G2NaDTaDTb (Fig. 2A). The immunogenicity and protective efficacy of these constructs in cotton rats were compared with BBG2Na and G2Na alone. Cotton rats received *im* 3 doses of 6 µg equivalent of G2Na in Alum: G2Na, BBG2Na, G2NaDTa, G2NaDTb, G2NaDTaDTb (Fig. 3A protocol). For simplicity, only ELISA IgG titres at day 60 (1 month after the 3^rd^ injection) are shown (Fig. 3B & 3C). Ab responses induced against G2Na or RSV-A were similar between the 5 groups after the 3^rd^ dose. The 2^nd^ dose induced similar anti-RSV-A Ab titres to the 3^rd^ dose (not shown). Importantly, lungs from cotton rats immunised with G2Na, BBG2Na or G2NaDTb were fully protected (virus at or below the limit of detection) against RSV-A challenge (Fig. 3D). Furthermore, mean lung virus titres in groups immunised with G2NaDTa or G2NaDTaDTb were also consistent with protection, while 3/6 cotton rats in both groups had no detectable virus. These data indicate that G2Na alone is highly immunogenic and protective against RSV-A challenge in cotton rats. Surprisingly, they also suggest that the BB or DT-derived carrier proteins do not enhance the immunogenicity or protective efficacy of G2Na in this model.

### Heterologous anti-RSV-A and RSV-B Ab responses induced by G fragment constructs in mice

RSV strains are separated into the A and B subgroups based primarily on the G protein sequence. Indeed, there is limited antibody cross-reactivity between native G proteins from each subgroup. We previously demonstrated that BBG2Na induced efficient but short term protective immunity against RSV B challenge in mice [34]. In an attempt to improve heterologous protective immunity, we constructed and expressed G2Nb, the RSV B (strain 8/60) G protein equivalent of G2Na, separately or fused to G2Na (G2ab) (Fig. 2B). G2Nb incorporates the 4 conserved Cys res. (see alignment Fig. 1B). The G2ab fusion protein contains G2Na (4 Cys) and the B component of G2 modified to contain only 2 Cys (Fig. 2B). Cys173 and Cys186 were substituted by Ser res. in the B component G fragment in order to limit the number of Cys res. in the fusion protein G2ab and to minimise spurious disulfide bonds between Cys res.. The rationale for this approach was provided by Simard *et al.* [34], who reported that a subgroup B G peptide with only 2 conserved Cys176 and Cys182 (peptide B174187.S186) induced protective immune responses against RSV-B.

**Figure 4:**
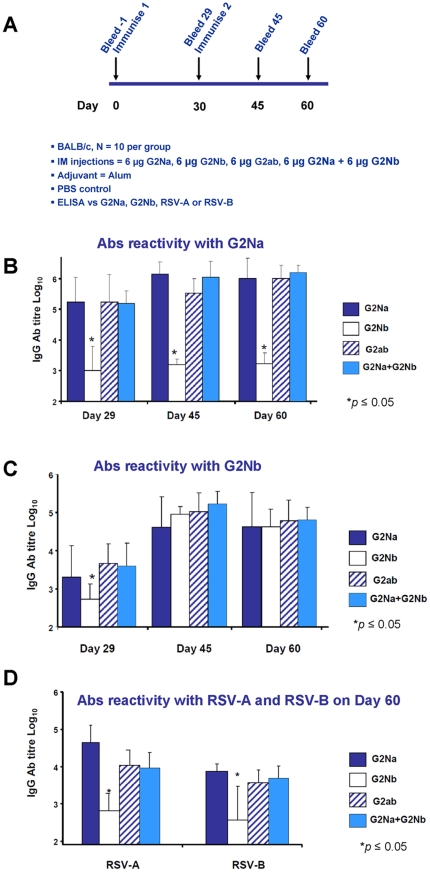
Immunogenicity of G2Na, G2Nb, G2ab and G2Na+G2Nb in mice. Groups of animals were immunised *im* twice with Ag/Alum according to experimental protocol outlined in (A). We determined ELISA IgG Ab titre induced respectively by G2Na (dark blue), G2Nb (white), G2ab (stripe) and G2Na+G2Nb (light blue) on D29, D45 and D60, against (B) G2Na (C) G2Nb and on D60 (D) RSV-A (Long) on the left panel and RSV-B (8/60) on the right panel.

We determined the immunogenicity of G2Na and G2Nb. In addition, we determined whether G2Na admixed with G2Nb or G2ab improved the Ab reactivity with RSV-B. (Fig. 2B). Groups of naïve mice were immunised *im* twice with 6 µg of each of the following antigens formulated in Alum: G2Na, G2Nb, G2ab, G2Na admixed with G2Nb, according to the protocol in Fig. 4A. Similar IgG antibody titres against G2Na were found in all groups, except in the G2Nb-immunised mice (Fig. 4B). In the other groups, high anti-G2Na Ab responses were evident after a single immunisation. Furthermore, mean antibody titres were boosted after the second dose. In contrast, the second injection of G2Nb did not boost Ab reactivity with G2Na, which was rather weak (≤3log_10_) compared to the other groups (*p*≤0.05). This might be explained by the fact that G2Nb shares only 52.5% sequence homology with G2Na (see alignment of Fig. 1B).

After the first injection, G2Na induced higher mean Ab reactivity with G2Nb in mice than G2Nb (Fig. 4C). After the second injection, on day 45, every immunised group had equivalent Ab reactivity with G2Nb that remained stable until day 60. Surprisingly, the presence of the subgroup B G fragment, either in the genetic fusion form G2ab or admixed with G2Na, did not boost the Ab reactivity with G2Nb.

On day 60, high levels of Ab reactivity with RSV A were evident in all mice immunised with G2Na alone, admixed with G2Nb or fused in G2ab (Fig. 4D). In contrast, G2Nb-immunised mice developed significantly lower RSV-A Ab reactivity, consistent with the poor G2Na Ab reactivity described above. Ab reactivity with RSV-B was also evident in each of these groups in similar relative amounts, although they were approximately 0.5 log_10_ lower than the anti-RSV-A Ab titres. Again, G2Nb was poorly immunogenic and Abs did not react with RSV-B, suggesting that the G2Nb fragment was misfolded. Collectively, these data showed that its presence did not boost Ab reactivity with RSV-B when associated with G2Na.

### Immunogenicity and protective efficacy of G2Na against both RSV-A and B strains

**Figure 5:**
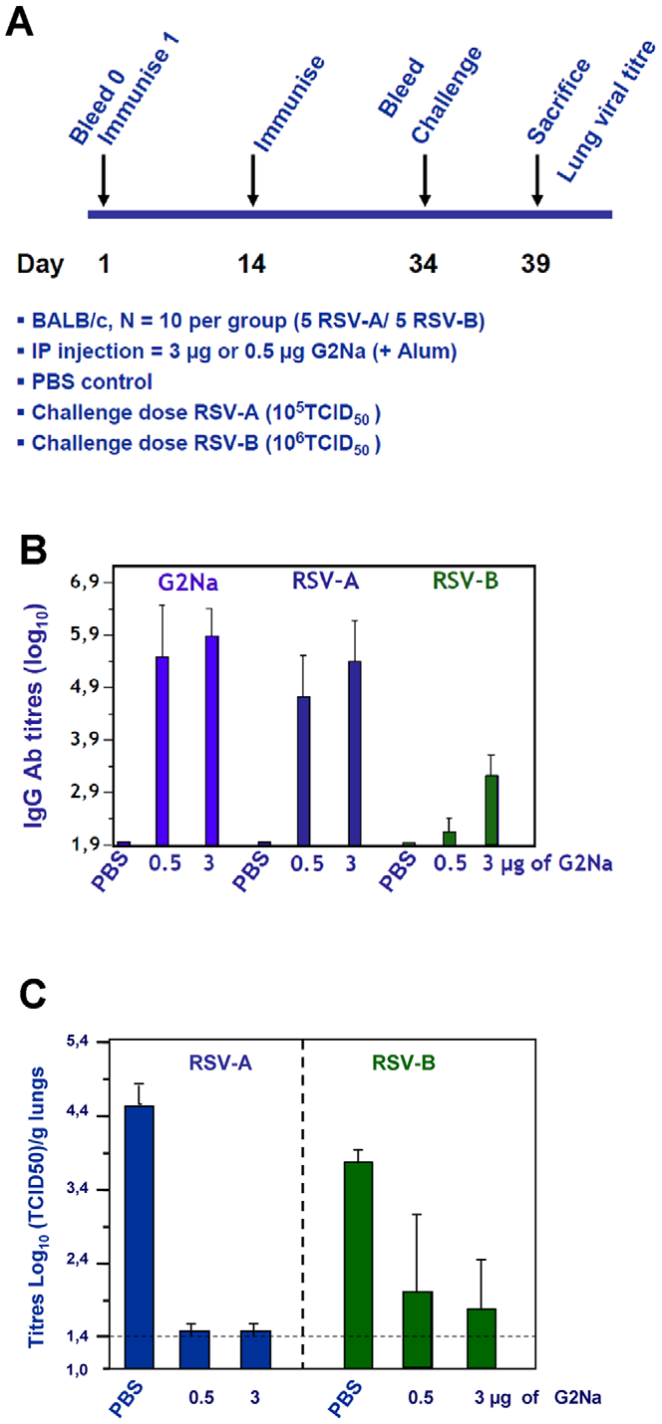
Immunogenicity and protective efficacy conferred by G2Na against heterologous challenge in mice. Groups of mice were immunised *ip* twice with either 0.5 µg or 3 µg of Ag in Alum according to the experimental protocol outlined in (A). After the 2^nd^ injection, we determined (B) by ELISA IgG Ab titre against G2Na (light blue) left panel, RSV-A (dark blue) middle panel and RSV-B (green) right panel. Immunised mice were divided into 2 groups (n = 5), before challenge with either RSV-A (10^5^ TCID_50_) and RSV-B (10^6^ TCID_50_). Protective efficacy was evaluated (C) by determining the titre in the lungs (expressed as Log_10_ TCID_50_/g lungs) of RSV-A (left panel, in dark blue) and RSV-B (right panel, in green). Dotted line represents the limited detection of RSV in the lungs.

To determine the capacity of G2Na to induce protective immunity against both RSV-A and B challenges in mice, naïve BALB/c mice were immunised *ip* with 2 doses of either 0.5 µg or 3 µg of G2Na with a 2 week interval (Fig. 5A). At day 34, half of the group (5/10) was challenged with RSV-A, while the other half was challenged with RSV-B. After the second injection, high Ab titres were detected in both the G2Na and RSV-A ELISAs (Fig. 5B). Importantly, these titres, particularly in the groups immunised with 3 µg G2Na, were consistent with those reported in Figs. 4B and 4D, respectively. Also consistent with Fig. 4D, Ab titres against RSV-B were lower than titres against G2Na or RSV-A. While mean Ab titres against G2Na and RSV-A suggest a slight dose effect, the differences were not significant. After homologous challenge with RSV-A, there was no detectable virus present in the lungs of G2Na-immunised mice, irrespective of the dose (Fig. 5C). After heterologous challenge of 3 µg G2Na-immunised mice with RSV-B, there was also no virus detected in 4/5 mice. However, virus was detected in the 5^th^ mouse. The mean titre of all 5 RSV-B challenged mice is plotted in Fig. 5C. While a significant reduction in lung titres of RSV-B was evident in mice immunised with 0.5 µg G2Na compared to controls, 3/5 mice had detectable virus in their lungs and the mean titre was above the cut-off for protection (>2log_10_ reduction). This is consistent with a study in which animals immunised thrice *ip* with 0.2 or 20 µg of BBG2Na demonstrated good protective immunity against RSV-B with the higher dose but less so with the lower dose [35].

### Duration of protective immunity induced by G2Na in cotton rats

**Figure 6:**
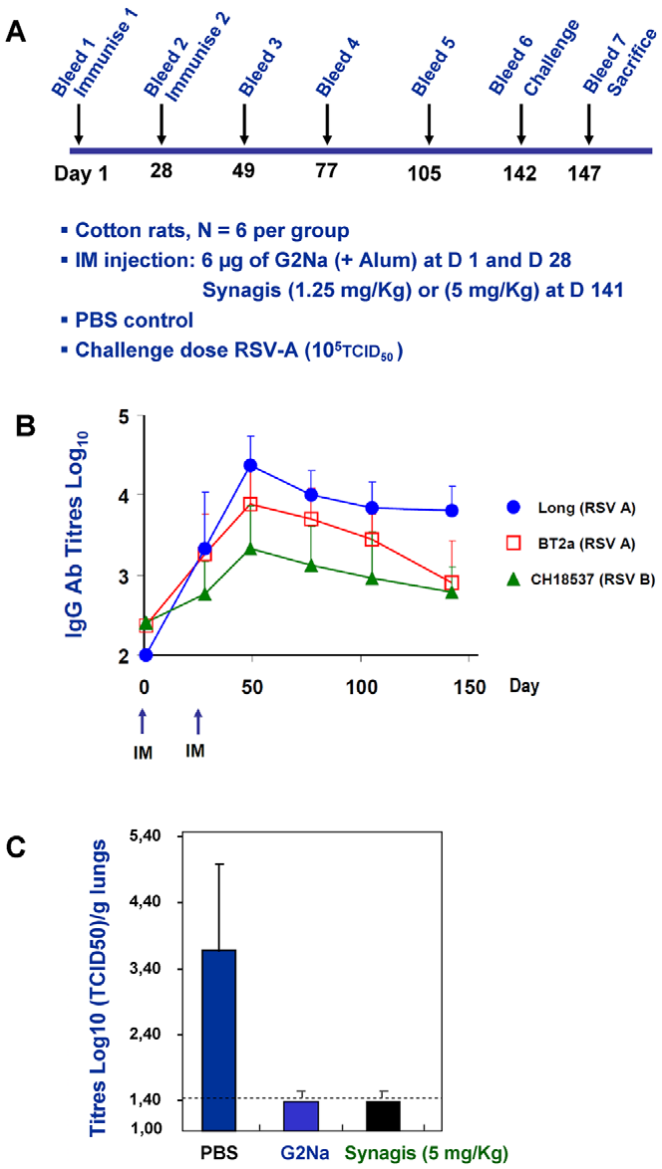
Persistence of Ab induced by G2Na in cotton rats and protective efficacy against RSV-A. Groups of animals were immunised *im* twice by Ag/Alum according to the experimental protocol outlined in (A). We studied the duration of Ab induced (B) by determining IgG Ab titre up to D148 against RSV-A Long strain (blue circle), RSV-A BT2a clinical strain (red square) and RSV-B CH18537 strain (green triangle). Protective efficacy of G2Na Ab induced against RSV-A challenge (10^5^TCID_50_) was evaluated in comparison to Synagis® (C) by determining titre (expressed as Log_10_ (TCID_50_)/g lungs titre of immunised and treated animals. From left to right: PBS control immunised group, G2Na immunised group and Synagis® (5 mg/Kg) treated group.

An important consideration for an RSV vaccine is that it is capable of inducing protective immune responses that persist for longer than the normal epidemic RSV season, i.e., ∼5 months. To address this, groups of cotton rats received 2 *im* injections of 6 µg G2Na. The kinetics of IgG responses was measured by ELISA against 3 RSV strains: Long (RSV A) from which G2Na peptide sequence was derived, BT2a (RSV A) a recent clinical isolate and CH18537 (RSV-B). As positive controls for Ab-mediated protection, 2 animal groups received *im* respectively 1.25 mg/Kg and 5 mg/Kg Synagis® one day before RSV A challenge (Fig. 6A protocol). The second injection of G2Na resulted in a significant boost in Abs reactive with anti-RSV-A Long (blue circle), which peaked at day 49 and declined slowly until day 148. It is noteworthy that the kinetics of RSV-A BT2a reactivity (red square) paralleled those of RSV-A Long until day 105, albeit at slightly lower titres, but appeared to decline more rapidly by the last point, day 148. As expected, based on the data presented in Figs. 4D and 5B, the kinetics of, and peak, Ab reactivity with RSV-B CH18537 (green triangle) were lower than the reactivity with RSV-A. Nonetheless, reactivities to RSV-B persisted for the duration of the experiment. Challenge at day 142 with infectious RSV-A (Long), produced no infectious virus in the lungs of G2Na-immunised cotton rats when assessed 5 days later (Fig. 6C). This protective efficacy compared favourably with positive control naïve cotton rats that received Synagis® prophylaxis (5 mg/kg) 1 day before challenge. The animal group receiving a lower dose (1.25 mg/Kg) was partially protected (RSV-A titre = 1.58±0.21 log_10_ TCID50. Virus was detected in 3/6 lung homogenates). Therefore, two injections of G2Na/Alum in cotton rats were sufficient to induce persistently elevated Ab responses capable of recognizing both RSV-A and, to a lesser extent, RSV-B for at least 21 weeks. Importantly, G2Na also induced persistent protective immunity against RSV-A in cotton rats.

### Induction of Ab responses in RSV primed mice by G2Na/Alum and G2Na/PLGA and Ab affinity maturation

In the context of the annual autumn Flu vaccine, it would be ideal to administer an RSV vaccine to the elderly at the same time as the influenza virus vaccine, as RSV and influenza virus are the most important viral pathogens causing severe RTI in this population and both circulate in the winter time [1], [2], [37]. In an attempt to model vaccine responses in RSV seropositive elderly, we addressed the immunogenicity of G2Na in RSV-A-primed mice, in which G2Na adsorbed to Alum (G2Na/Al) was compared to G2Na encapsulated in PLGA (Fig. S1). We also examined the avidity of Ab induced against RSV-A over time. RSV-A-primed mice were injected *im* with a single dose of G2Na encapsulated in PLGA. The aim was to have intermittent intra-corporal release of antigen over a period of months that might boost RSV-specific immune responses and thereby maintain elevated antibody titres over a longer period.

**Figure 7:**
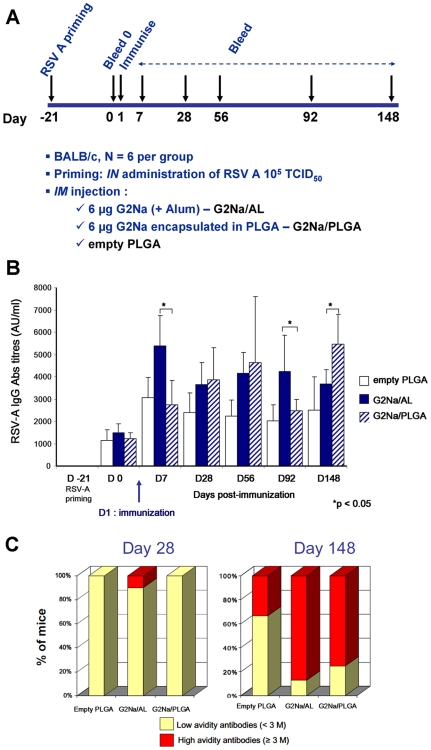
Persistence and affinity maturation of Ab induced by G2Na/AL and G2Na/PLGA in RSV-A primed mice. Groups of primed-mice were immunised *im* once according to the experimental protocol outlined in (A). We monitored Ab induction on D0, D7, D28, D56, D92 and D148 by ELISA IgG (B) anti-RSV-A IgG induced by empty PLGA (white bars), by G2Na/AL (blue bars), and by G2Na/PLGA (striped blue bars). Results are expressed as Arbitrary Units (AU)/ml using a positive control serum as a reference. * p<0.05. We determined affinity maturation of each serum Ab (see Materials and Methods) and summarised it in (C) bar graphic representation of % of animals with low avidity Ab (<3 M) in yellow and with high avidity Ab (≥3 M) in red on D28 (left panel) and on D148 (right panel). In the panels from left to right: empty PLGA group, G2Na/AL group and G2Na/PLGA group.

Mice were primed by *in* administration of 10^5^ TCID_50_ RSV-A. Three weeks later, sera were screened for presence of anti-RSV Ab before *im* injection (Fig. 7A). RSV-A-primed mice were injected *im* with G2Na/AL, G2Na/PLGA or control empty PLGA. In the control empty PLGA group (no antigen added), RSV-A Ab levels (white bar) increased after the priming and peaked at day 7 post-vaccination but the increases were not significant. G2Na/AL rapidly and significantly boosted the RSV-A Ab reactivity (black bar), which then slowly declined. On D148, Ab titres were not significantly different from those of mice receiving empty PLGA. In contrast, G2Na/PLGA induced a biphasic increase in RSV-A titres: a first peak observed on D56, and a second one on D148 (striped bar). At this time point, RSV-A titres were significantly higher compared to both G2Na/AL- and empty PLGA-immunised mice. Those kinetics probably reflected the intermittent release of G2Na from PLGA microspheres. Although the kinetics of *in vivo* release of Ag protein was unknown, our hypothesis is that: the first Ab peak response was due to diffusion of PLGA surface-absorbed G2Na protein and the second Ab peak response was mainly due to complete hydrolysis of the polymer which released all the encapsulated G2Na Ag. Minor continuous release of Ag might happen in between. This type of release was difficult to standardise as was described with microencapsulated tetanus toxoid vaccine [31]. Further studies with different formulations (different type of PLGA size) should be conducted in order to optimise the appropriated burst release ot the Ag.

We also examined the RSV-A-specific avidity of IgG following a single injection of either G2Na/AL or G2Na/PLGA in RSV-A primed mice (Fig. 7C). The avidity was determined by ELISA elution using ammonium thiocyanate (NH_4_SCN) as chaotropic agent, as described [33], [49]. Results are expressed as the avidity index (AI), the concentration of thiocyanate required to elute 50% of Ab, and as proportions of high-avidity, which was defined for this antigen as the proportion of Ab molecules that remain bound to the plates at NH_4_SCN concentration ≥3 mol/L (≥3 M). For clarity we represented the % of mice in each immunised group having low (<3 M; yellow) and high (≥3 M; red) avidity, respectively at days 28 (left panel) and 148 (right panel) post-immunisation with G2Na. One month after G2Na immunisation, almost all mice had low avidity RSV-A antibodies. In contrast, by day 148 post G2Na immunisation, either adsorbed to Alum or encapsulated in PLGA microspheres, elicited a dramatic increase in high avidity RSV-A antibodies.

## Discussion

In this study, we have comprehensively addressed a number of important issues relating to the use of a recombinant protein incorporating the central conserved domain of the RSV G protein as a subunit RSV vaccine. First, we demonstrated that a carrier protein fusion partner, in the form of DTa, DTb or BB, did not enhance the immunogenicity or protective efficacy of G2Na against homologous virus challenge. This constrasts with our previous work, in which BBG2Na was found to potentiate anti-G2Na Ab responses compared to G2Na alone [27]. Different routes of antigen administration (subcutaneous versus *im* or *ip*) might account for these discrepancies. In the current study, the capacity of *ip* or *im* G2Na, formulated in Alum, to induce protective immunity against an RSV-A challenge was unambiguously demonstrated in 2 animal models, mice and cotton rats.

As RSV strains are divided into subgroups based primarily on G protein and gene heterogeneity, it was important to address the capacity for induction of heterosubtypic protective immunity by RSV-A- and/or B-derived G fragments. Surprisingly, G2Na was considerably more immunogenic compared to its G2Nb counterpart at inducing Abs reactive with both RSV-A and B. Furthermore, genetically fusing or admixing the respective RSV-A and B G2N antigens did not enhance these responses. The poor immunogenicity of G2Nb contrasts with the work of Simard *et al* [36], who showed that the peptide B174187.S186, corresponding to amino acid res. 174–187 of RSV-B G, induced protective anti-RSV-B Ab responses. The B174187.S186 peptide contains the 2 conserved Cys res. (Cys176-Cys182), while the third Cys186 was substituted by Ser. It is noticed that the corresponding native G peptide 174–187 contains only 3 Cys, the substitution of the Cys186 to Ser prevented uncorrected disulfide bonds. Furthermore, Murata et al. [38] recently showed that the central domain of G from both RSV subtypes (aa151–190) had high reactivity to sera from adults infected with RSV-A or B and they suggested that peptides from either the A or B strains might be sufficient to elicit viral subtype-independent Ab production and protective efficacy. The poor immunogenicity and protective efficacy of G2Nb might be explained by inappropriate protein folding, although this remains to be proven. While the level of Abs induced by G2Na reacted in a dose-dependent manner with both RSV-A and RSV-B, the reactivity with RSV-B was clearly inferior. Thus, the possibility remains that RSV-B reactivity following G2Na vaccination may be insufficient to protect from RSV-B. G2Na dose-optimisation experiments will be required to maximise such responses. The series of proteins or peptides of RSV-B should be redesigned in order to get the appropriated conformational “protectopes”.

The native F protein is another potential candidate to be considered because its aa sequence is highly conserved between A and B strains. However, extraction and purification of F protein from infected eukaryotic cells that are appropriate for human vaccine use are still problematic for industrial development [39], [40]. Recently, the production of recombinant F protein (with no transmembrane and no cytoplasmic tail) in a soluble pretriggered form from 293T mammalian cells was described [41]. Whether or not this may be scaled up to industrial production remains to be determined, as does the protective efficacy and immunogenicity of the final product.

The duration of protective immunity following vaccination is another critical parameter for any vaccine. Our anti-RSV-A Ab responses and protection against homologous virus challenge, are promising on this account. The duration of the experiment (148 days) corresponds with an entire RSV season in temperate zones [3]. The persistence of strong protective immunity in the cotton rat model for this period suggests that the G2Na/Alum formulation might constitute a suitable vaccine for humans. The fact that elevated Ab reactivity to a recent RSV-A clinical isolate and RSV-B also persisted for the duration of the experiment is also very encouraging.

In view of the devastating history of the Alum-adjuvanted formalin-inactivated RSV vaccine in infants in the 1960s [4], a subunit RSV vaccine is unlikely to be administered to infants that are immunologically naive to RSV without a comprehensive understanding of the mechanisms of the enhanced pathology. Although extensive animal studies demonstrated that BBG2Na did not induce FI-RSV-like immunopathology [22], [42], [43], [44], [45], the immunopathogenic potential of G2Na without the BB part remains to be determined. Increasing epidemiological evidence indicates that RSV infection causes considerable morbidity and mortality in the elderly [1], [36]. Because they have been repeatedly infected throughout life with RSV and have developed a pattern of safe immune responses, they should not be susceptible to the vaccine-mediated enhanced pathogenesis phenomenon described in young infants.

Ideally, an RSV vaccine should confer protection against both RSV-A and RSV-B strains. For this purpose, antigens originating from both strains might be required. Native Ag purified from RSV infected cells or recombinant eukaryotic expression (FG expressed in baculovirus) have been studied in clinical trials [8], [46], [47], [48]. However, native Ag purified from RSV infected mammalian cells in industrial scale still pose considerable problems with regard to yield and cost. Therefore, a recombinant vaccine, such as G2Na, produced in *E. coli* with high yield and low cost, may be sufficient and safe in this target population. As already described for lived vaccines [49], the presence of pre-existing anti-RSV Abs in this population may compromise such a vaccine. However, the data presented in Fig. 7 suggest that G2Na, formulated in either Alum or PLGA, is immunogenic in mice in the presence of pre-existing anti-RSV-A Abs. The persistence of the Ab responses for at least 148 days was also very encouraging. The significantly elevated anti-RSV-A Ab responses at the end of the experiment in mice immunised with G2Na/PLGA remains to be correlated to increased protective efficacy to demonstrate that this is a promising formulation. Importantly, we provide robust evidence that vaccination with different G2Na formulations in the presence of anti-RSV-Abs did not compromise avidity maturation of the Abs over time.

In conclusion, our study clearly demonstrated that G2Na has many characteristics appropriate for a vaccine antigen against RSV in the elderly. For the seronegative infant target population, it should be carefully monitored in terms of immunopathology in animal models. This promising Ag could be associated with an RSV-B G peptide component or F protein, and/or formulated with a specific immune response driving adjuvant (e.g., TLR agonists) to achieve both safety and protective efficacy against RSV.

## Supporting Information

Figure S1 **Encapsulation of G2Na in PLGA [poly(D,L-lactide-co-glycolide].** Schematic representation of the encapsulation as described in detail in Materials and Methods. The Ag microencapsulation efficiency was evaluated to 68%. On the upper right, we show micro photographs from electronic microscopy of encapsulated microspheres in order to evaluate the homogeneity of the recovered PLGA. Graph of distribution of size showed an average diameter of 14.8 µm.
(TIF)
