# Supplementary figures and images for: The Respiratory Syncytial Virus G Protein Conserved Domain Induces a Persistent and Protective Antibody Response in Rodents
Source: PLoS One. 2012 Mar 29;7(3):e34331. doi: 10.1371/journal.pone.0034331 (PMC3315535; doi:10.1371/journal.pone.0034331)

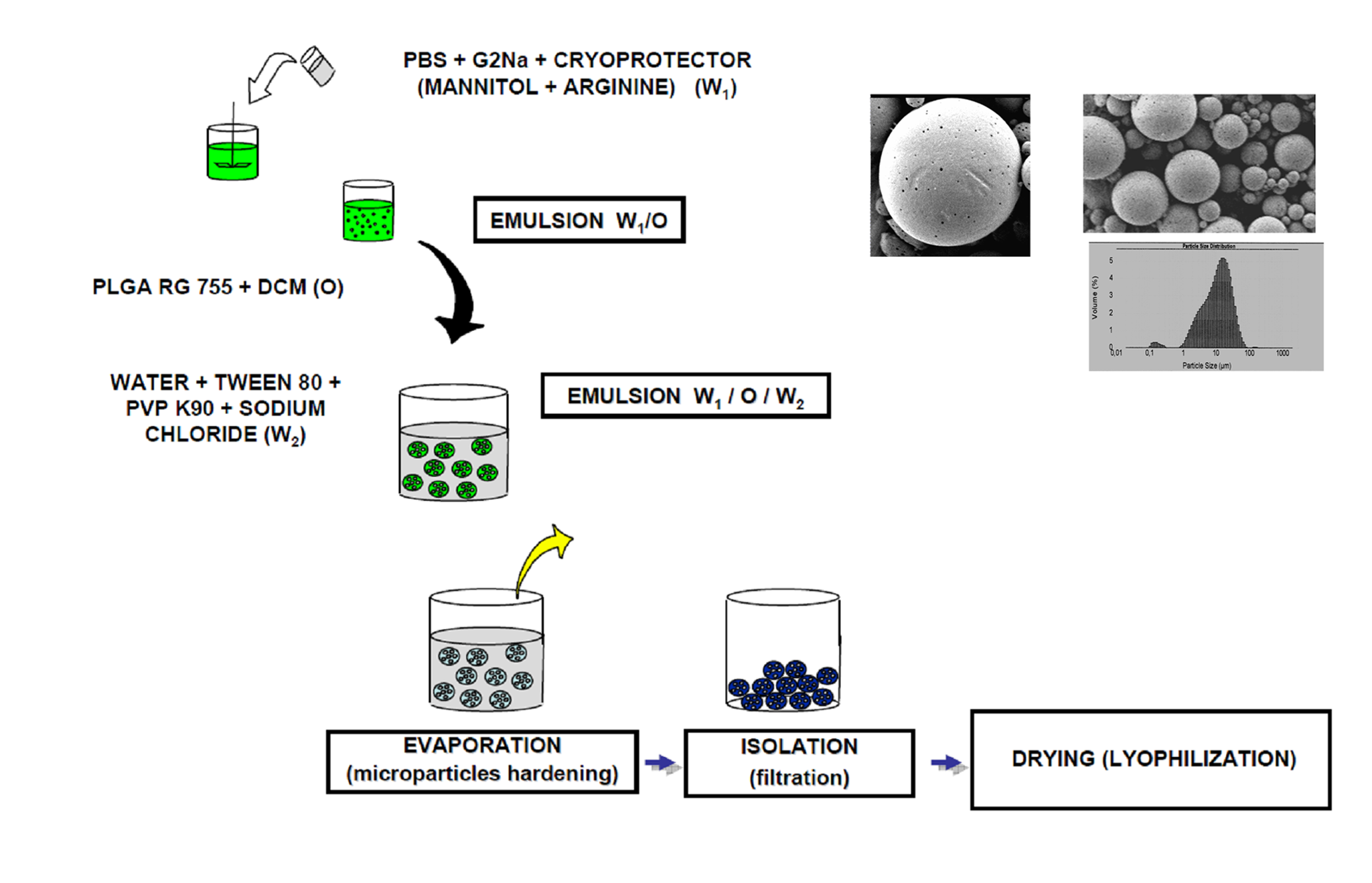

Supplement: Figure S1 — Encapsulation of G2Na in PLGA [poly(D,L-lactide-co-glycolide]. Schematic representation of the encapsulation as described in detail in Materials and Methods. The Ag microencapsulation efficiency was evaluated to 68%. On the upper right, we show micro photographs from electronic microscopy of encapsulated microspheres in order to evaluate the homogeneity of the recovered PLGA. Graph of distribution of size showed an average diameter of 14.8 µm. (TIF) [file pone.0034331.s001.tif]
